# Analysis of A 6-Mirna Signature in Serum from Colorectal Cancer Screening Participants as Non-Invasive Biomarkers for Advanced Adenoma and Colorectal Cancer Detection

**DOI:** 10.3390/cancers11101542

**Published:** 2019-10-12

**Authors:** María Marcuello, Saray Duran-Sanchon, Lorena Moreno, Juan José Lozano, Luis Bujanda, Antoni Castells, Meritxell Gironella

**Affiliations:** 1 Gastrointestinal and Pancreatic Oncology Research Group, Centro de Investigación Biomédica en Red de Enfermedades Hepáticas y Digestivas (CIBEREHD)/Hospital Clínic of Barcelona/Institut d’Investigacions Biomèdiques August Pi i Sunyer (IDIBAPS), University of Barcelona, 08036 Barcelona, Spain; 2 Bioinformatics Platform, CIBEREHD, 08036 Barcelona, Spain; 3 Department of Gastroenterology, Hospital Donostia/Instituto Biodonostia, CIBEREHD, Universidad del País Vasco UPV/EHU, 20014 San Sebastián, Spain

**Keywords:** colorectal cancer, screening, non-invasive biomarker, early detection, advanced adenoma, circulating miRNA, serum, liquid biopsy

## Abstract

Early detection of colorectal cancer (CRC) and its precancerous lesion, advanced adenomas (AA), is critical to improve CRC incidence and prognosis. Circulating microRNAs (miRNAs or miR) are promising non-invasive biomarkers for cancer detection. Our previous results showed that a plasma 6-miRNA signature (miR-15b-5p, miR-18a-5p, miR-29a-3p, miR-335-5p, miR-19a-3p and miR-19b-3p) could distinguish between CRC or AA and healthy individuals (controls). However, its diagnostic performance in serum is unknown. In this exploratory study we aim to evaluate the diagnostic performance of the 6-miRNA signature in serum samples in a cohort of individuals participating in Barcelona’s CRC Screening Programme. We prospectively collected serums from 264 faecal immunochemical test (FIT)-positive participants and total RNA was extracted. Finally, 213 individuals (CRC, 59, AA, 74, controls, 80) were included. MiRNA expression was quantified by real-time RT-qPCR and data analysis was performed by logistic regression. Faecal hemoglobin concentration (f(Hb)) from FIT of the same individuals was also considered. As previously described in plasma, serum from patients with AA or CRC presented significant differences in the 6-miRNA signature compared to controls. Moreover, when combined with f(Hb), the final signature showed high discriminative capacity to distinguish CRC from controls (area under the curve (AUC) = 0.88), and even AA (AUC = 0.81) that otherwise are poorly detected if we only consider f(Hb) (AUC = 0.64). Addition of the serum 6-miRNA signature to quantitative f(Hb) show high accuracy to detect patients with advanced colorectal neoplasia in average-risk individuals. A combination of these two non-invasive methods could be a good strategy to improve diagnostic performances of current CRC screening programmes.

## 1. Introduction

Colorectal cancer (CRC) is the most frequent cancer in Western countries and the second leading cause of cancer-related death [1]. The global patient’s survival gets worst with the increase of tumoral stage at the moment of diagnosis, considering CRC as an important target of screening programmes. Indeed, almost 90% of CRC patients who are detected in an early stage survive, whereas only 12–13% of patients survive when detected with metastatic disease [2,3]. It is well known that CRC is preceded by the development of pre-malignant lesions—mainly advanced adenomas (AA)—which can be detected and excised, preventing their progression towards malignant tumour lesions. Therefore, precancerous AA represents a pertinent target lesion for a CRC screening test.

Although some strategies are available to screen CRC average-risk individuals including colonoscopy or non-invasive faecal occult blood tests, such as the faecal immunochemical test (FIT), each one of them have important disadvantages. First, colonoscopy is the main method to detect both CRC and AA, but it is invasive, it is not exempt from complications for the patient, it requires patient preparation and it is expensive. On the other hand, FIT is non-invasive and shows quite accurate results in CRC detection but is compromised by low sensitivity (Sn) in detecting AA and a high ratio of false positive results [4]. Therefore, early detection of AA remains an unmet need since there is not an available test with promising diagnostic performance for these lesions. Although a combination of stool DNA and occult blood testing has recently emerged as a potential useful approach to overcome this low sensitivity on AA detection [5], it is associated to low specificity, high cost and logistic difficulties due to the large amount of fresh faeces needed, thus making it difficult to be used in a population-based scenario. Moreover, adherence to stool-based screening is partly compromised due to the kind of sample that people need to handle. Therefore, a blood-based test with high diagnostic performances for both CRC and AA would offer advantages compared to the current available strategies.

During the last decade, circulating microRNAs (miRNAs or miRs) have emerged as new promising non-invasive biomarkers for CRC early detection due to their high stability in biofluids [6,7] and their easy detection in low quantities of sample. Previous results from our group showed that several miRNA members of the oncogenic miR-17-92 cluster [8] together with other miRNAs (miR-15b-5p, miR-18a-5p, miR-29a-3p, miR-335-5p, miR-19a-3p and miR-19b-3p), were significantly upregulated in plasma from patients with CRC or AA, compared to control subjects [9]. Subsequent studies have confirmed these results in a large cohort of plasma samples [10]. Interestingly, most of the individual miRNAs that make up this 6-miRNA signature have also been found to be deregulated in serum or plasma samples from CRC patients by others [11,12,13,14,15,16,17]. It is important to test a biomarker in different body fluids in order to demonstrate its robustness as a biomarker, as well as to unveil the best strategy for its analysis. However, the diagnostic performance of this 6-miRNA signature in another widely used biofluid such as serum is still unknown.

Therefore, the aim of this study was to further evaluate the previously described 6-miRNA plasma signature [9,10] in serum samples from a large cohort of FIT-positive individuals coming from a CRC screening programme. Moreover, a combination of different non-invasive strategies could improve CRC early detection, and better understanding the behaviour of these biomarkers in different biofluids and different cohorts of patients will boost its translation into the clinic.

## 2. Materials and Methods

### 2.1. Patients and Samples

**Figure 1:**
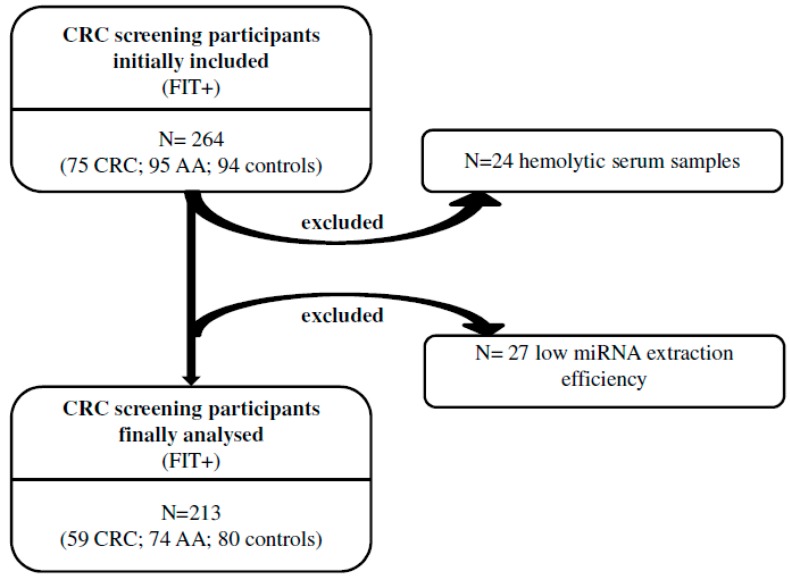
Schematic representation of the study. Two-hundred and sixty-four serum samples were collected from individuals positive to the faecal immunochemical test (FIT+) and after colonoscopy were distributed in three groups: colorectal cancer (CRC), advanced adenomas (AA) and individuals with normal colonoscopy (controls). After serum haemolysis assessment and microRNA (miRNA) extraction and spike-in cel-miR-39 quantification, 51 samples were discarded from the final analysis.

A total of 264 FIT-positive participants in Barcelona’s CRC screening programme were recruited for a restrospective, case-control study (94 healthy subjects with no history of any cancer, controls, 95 patients with AA and 75 patients with CRC). Peripheral blood samples were prospectively collected before colonoscopy and before any polyp or cancer resection and centrifuged after coagulation at 1500 g for 10 min to obtain serum samples. Once obtained, serum samples were stored at −80 °C until use. Finally, 51 samples were discarded for different reasons, as detailed in Figure 1, and the final number of patients included was 213. Characteristics of these patients are shown in Table 1. The CRC staging system used was the American Joint Committee on Cancer Tumour Nodes Metastasis classification. The study was approved by the Hospital Clinic of Barcelona’s Ethical Committee (ethical code: 2013/8110), and all participants provided written informed consent according to the Declaration of Helsinki.

### 2.2. Hemolysis Determination

Sample haemolysis can affect plasma and serum miRNA levels due to miRNA contamination coming from blood cells [18]. To check our samples for in a preanalytical phase before RNA extraction, serum absorbance was measured spectrophotometrically using Epoch (Biotek Instruments, Winooski, VT, USA). Absorbance at 414 nm and 375 nm wavelengths was measured to identify the presence of free haemoglobin and lipid content, respectively [19]. Since the measure at 414 nm is not fully representative of the level of haemolysis due to possible interferences from lipid content, the ratio between both values (absorbance 414 nm/absorbance 375 nm) was calculated to normalize the 414 nm value [20]. Then, samples with an absorbance ratio 414/375 nm > 2, were removed from the study, and considered as haemolysed. Dubious samples were confirmed by quantifying expression of the haemolysis related miR-451 by qRT-PCR [19]. In the end, 24 serums samples were discarded for showing an absorbance ratio 414/375 nm > 2 and high levels of miR-451.

### 2.3. RNA Extraction

Total RNA was isolated from 500 μL of serum (the same volume for all the patients) by using miRVana PARIS Kit (Ambion by Thermo Fisher Scientific Inc., Foster City, CA, USA) following the manufacturer’s protocol. For all the samples, 5 μL of cel-miR-39 spike-in (5 nM) were added during the initial steps of RNA extraction, as an exogenous control for normalisation of technical variations between samples. The elution volume with RNase-free water was 30 μL. Finally, 27 samples were discarded due to the extremely high Ct value of the spike-in miRNA since it means a low miRNA extraction efficiency.

### 2.4. Analysis of miRNA Expression by Real-Time qRTPCR

MiRNA expression was assessed by singleplex qRT-PCR with preamplification using TaqMan miRNA Assays (Thermo Fisher Scientific Inc.), according to the manufacturer’s protocol. Briefly, six target miRNAs (miR-29a-3p, miR-15b-5p, miR-18a-5p, miR-19a-3p, miR-19b-3p, miR-335-5p) and two control miRNAs (miR-1228 as endogenous control and cel-miR-39 as exogenous control) were assessed in all samples, as previously described [10]. The amount of input RNA per reaction was standardized by extracting RNA from exactly the same serum volume for all patients, and analysing the same volume of RNA per reaction. Pre-amplification was carried out for 12 cycles and real-time PCR was run in a Viia7 Real-Time PCR System (Applied Biosystems, Thermo Fisher Scientific Inc.). Each PCR reaction was performed in triplicate. Ct data were normalised by the geometric mean of cel-miR-39 and miR-1228 as house-keepings.

### 2.5. Faecal Haemoglobin (f(Hb)) Determination

Faecal haemoglobin concentration (f(Hb)) values (ng/mL) of the same patients participating in Barcelona’s CRC screening programme were obtained from FITs analysed with the automated FIT system OC-Sensor (Eiken Chemical Co., Tokyo, Japan).

### 2.6. Statistical Analysis

Fold change (FC) values were transformed to a log_2_ scale and a *t*-test was applied. False discovery rate (FDR) was computed using Benjamini and Hochberg procedure (FDR < 0.05 is considered as significant). Evaluation of predictability of individual or combined miRNAs was calculated using multivariate logistic regression adjusted by age and gender. Receiver operating characteristic (ROC) analysis plots and derived cut-points, as well as overall discriminative accuracy parameters, were computed using pROC R-package [21] (https://cran.r-project.org/web/packages/pROC/index.html) considering each miRNA expression or f(Hb) concentration as a continuous variable. Sn and specificity (Sp) were calculated from the cut-point associated with a Sn around 80%. We considered *p*-value < 0.05 as statistically significant.

## 3. Results

Out of a total of 264 FIT-positive individuals enrolled, 213 were successfully evaluated considering exclusion of 24 for presenting serum haemolysis, and 27 due to low miRNA extraction efficiency. Out of these 213, 80 were considered controls after colonoscopy, 74 AA and 59 CRC. In more detail, we enrolled 150 (70%) men and 63 (30%) woman within the age range of the CRC screening programme (50–69 years old). Mean age in all groups was between 61 and 62 (Table 1).

The AA group included subjects with at least one of the following characteristics: adenoma size bigger than 10 mm, high grade of dysplasia and/or villous component. The CRC group mostly included early stages (73%), stage I being the most abundant with 51% of the total. With respect to location, distal tumours were the most represented (69.5%).

### 3.1. A Signature Combining 6-miRNAs in Serum and f(Hb) Show High Accuracy for Detecting Patients Harbouring Advanced Adenomas (AA) or Colorectal Cancer (CRC)

Individually, serum samples from subjects with AA presented a significant increase of miR-29a-3p, miR-19a-3p and miR-335-5p in comparison to control ones. Among those, miR-19a-3p and miR-335-5p were also significantly upregulated in CRC serum samples, whereas upregulation of miR-29a-3p did not reach statistical significance (*p* = 0.07) in this cohort of patients (Supplementary Table S1).

**Figure 2:**
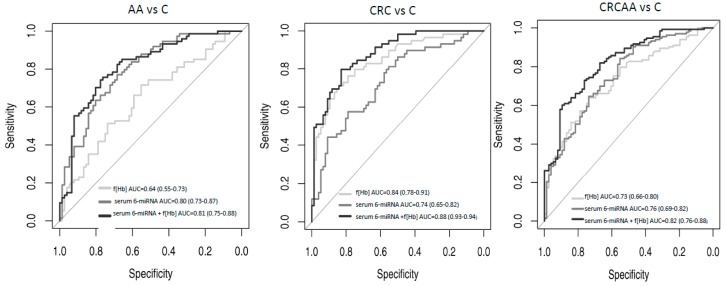
Receiver operating characteristic (ROC) curves of a serum 6-miRNA signature (miR-19a-3p + miR-19b-3p + miR-335-5p + miR-29a-3p + miR-15b-5p + miR-18a-5p) adjusted by age and gender, based on qRT-PCR results. Illustrating the FIT value alone, the 6-miRNA signature alone, the faecal haemoglobin concentration (f(Hb)) alone and the combination of 6-miRNA signature+ f(Hb). Left: patients with advanced adenomas (AA) versus controls, middle: colorectal cancer (CRC) patients versus controls, right: patients with advanced colorectal neoplasia (CRC or AA) versus controls.

Furthermore, the signature combining the 6-miRNAs in serum showed a very high discriminative capacity between AA and C patients with a ROC curve of area under the curve (AUC) = 0.80 (Figure 2 and Table 2). Surprisingly, the capacity of the 6-miRNA signature to distinguish CRC from control patients was less accurate with a ROC curve of AUC = 0.74. However, when adding f(Hb) values to this signature the diagnostic performances improved significantly, mainly for CRC. Thus, ROC curves for CRC achieved an AUC = 0.88 and for AA an AUC = 0.81. These discriminative capacities were higher than the ones observed when only using f(Hb) in the same cohort of patients that were AUC = 0.84 and 0.64, respectively (Figure 2 and Table 2). Globally, the ROC curve of the signature combining serum expression of the 6 miRNAs and f(Hb) value showed an AUC = 0.82 to distinguish those patients that have to undergo colonoscopy (AA or CRC) from control ones. The corresponding AUC obtained only considering f(Hb) values was 0.73.

### 3.2. Diagnostic Performance of the Combined Signature Does not Depend on CRC Stage or Location

**Figure 3:**
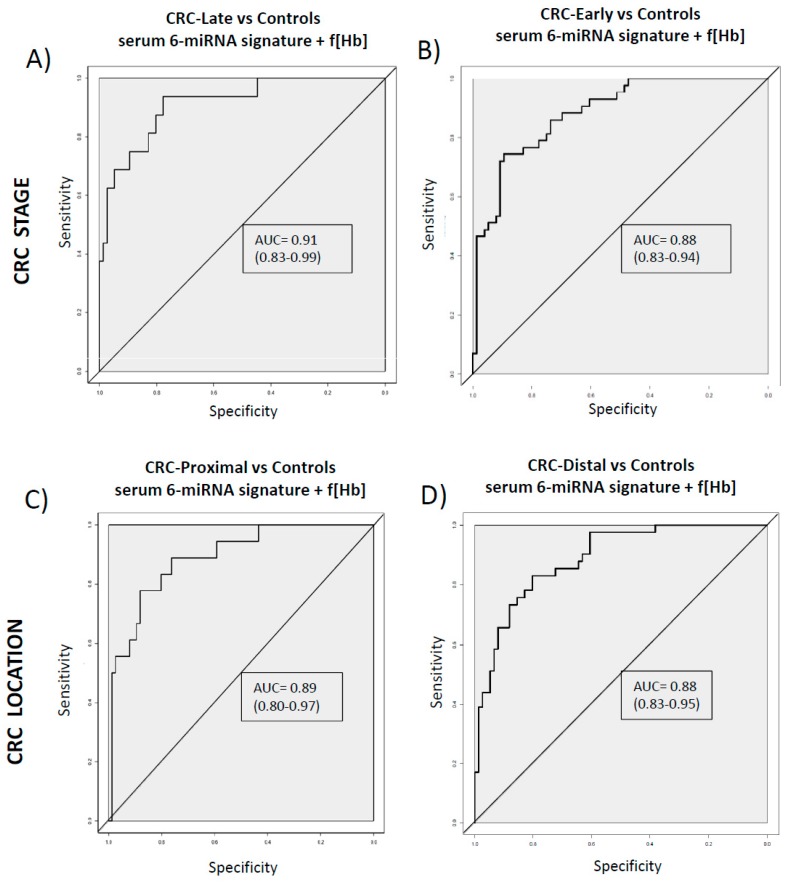
ROC curves of the serum 6-miRNA signature + f(Hb) adjusted by age and gender in CRC patients from different stages and different locations. (**A**) CRC Late stage (including III/IV). (**B**) CRC Early stage (including I/II). (**C**) CRC Proximal (including the ascending colon, the cecum, the right side of the colon and the transverse colon). (**D**) CRC Distal (including descending colon, left side of the colon, sigmoid colon, and the section of the colon that connects to the rectum). Colorectal cancer (CRC), controls (C), faecal haemoglobin concentration (f(Hb)).

Furthermore, the discriminative capacity of the signature combining serum 6-miRNAs and f(Hb) values was similar when comparing CRC from late stages (III/IV) and CRC from early stages (I/II) versus control subjects (Figure 3A,B). Regarding CRC location, there are no differences in the capacity of distinguishing proximal or distal tumours in comparison to controls (Figure 3C,D).

## 4. Discussion

In this study, we explored the possibility of using a non-invasive miRNA signature in serum for CRC and AA detection. It is utterly important to move the CRC screening field forward, since CRC survival depends on the stage of the disease at the time of diagnosis, localised diseases having a five-year survival rate of 90%, compared to a 10% survival rate for patients with a metastatic disease at the moment of diagnosis [22]. Therefore, CRC is one of the tumours that can most benefit from population screening programmes. Currently, colonoscopy and FIT are the most used tests in this setting, but colonoscopies are invasive for patients, require an intensive time commitment (bowel preparation, procedure and recovery) and are expensive. On the contrary, FIT is non-invasive, but it shows a limited capacity to detect precancerous lesions such as AA, and presents a high rate of false positives that have to undergo unnecessary colonoscopies [23]. It is well known that minimally invasive tests will grow in popularity over time taking into consideration the availability, costs and patient’s clinical preferences. Finally, the necessity to improve the accuracy for CRC and AA detection requires more of an effort to be made in this domain.

It is important to highlight that in this study, we analysed our previously reported circulating 6-miRNA signature [9,10] in a different biofluid such as serum, and in a FIT-positive cohort of average-risk individuals coming from a CRC screening programme. Moreover, we compared the diagnostic performance of the 6-miRNA signature with the performance of using the quantitative FIT value alone, f(Hb), in the same cohort of patients, and finally with the performance of combining both non-invasive strategies.

Our results demonstrated that a combination of the serum 6-miRNA signature with f(Hb) show good discriminative capacity for detecting patients harbouring AA or CRC, higher than the one exhibited only considering f(Hb) alone. This improvement was especially important in the detection of AA which are poorly detected by f(Hb) and are ideal targets if we want to have an incidence in CRC prevention. Moreover, this combination can detect with similar accuracy early and late CRC stages and CRC tumours from different localizations. All that would offer superiority to FIT because some authors have described a better diagnostic performance for CRC in distal than proximal colon with the FIT [24]. This is quite important due to the incidence of right-sided colon cancers has been increasing in the last years [25] and colonoscopy is more effective to detect left-side compared to right-side [26].

The miRNA signature includes 6 miRNAs (miR-15b-5p, miR-18a-5p, miR-29a-3p, miR-335-5p, miR-19a-3p, miR-19b-3p) previously described and related to CRC development. Specifically in this study, the most upregulated in CRC or AA serum samples were miR-19a-3p, miR-335-5p and miR-29a-3p. Concretely, a recent publication gave insights about the implication of miR-19a-3p in CRC development, promoting cell proliferation and migration [27]. There are a few more studies that have already focused on the upregulation of miR-19a-3p in CRC plasma and serum samples [28], and it also has been described within the polycistronic cluster miR17-92 commonly amplified in CRC [11]. Huang et al., also described their implication in mediating epithelial to mesenchymal transition and metastatic behaviour in CRC [29]. On the other hand, miR-29a-3p has been suggested several times as a promising circulating biomarker for CRC early detection [14,16]. Finally, although the exact role of miR-335 in CRC is still unknown, it has been described that overexpression of miR-335 increases colorectal cancer cell proliferation and tumour growth [30].

The serum 6-miRNA signature combined with f(Hb) values showed a Sn of 81% and a Sp of 78% for CRC, and a Sn of 81% and Sp of 69% for AA, these values were better than using f(Hb) alone (Sn = 81%, Sp = 73% for CRC and Sn = 81%, Sp = 35% for AA, respectively). It is important to highlight that it is the first study analysing this 6-miRNA signature in serum samples but a precise comparison with the performance obtained in plasma from previous studies by our group [10] cannot be performed due to differences in cohorts’ characteristics. In order to carry out a reliable comparison of the miRNA signature performance in both biofluids, a paired study with both samples from the same patient should be done. However, what can be drawn from this study is that this 6-miRNA signature can also work in serum in the CRC screening setting and that, more importantly, it is the first time that this circulating 6-miRNA signature is compared with another non-invasive biomarker such as the f(Hb) showing its superiority for precancerous lesions, and combined with it to improve overall diagnostic performances.

Moreover, it is noteworthy that in this study all the serum samples were obtained from CRC screening participants and, therefore, the CRC cohort has a greater presence of early stages, mainly stage I. It is important to highlight a recent study also carried out in FIT-positive screening participants discovering and validating three miRNA signatures in plasma from patients with CRC, high-grade adenomas and low-grade adenomas, respectively [31]. Although differences may exist between serum and plasma miRNA profiles, as well as using different centrifugation speeds, it is important to mention that those signatures and ours share one miRNA (i.e., miR-335-5p).

However, if we think in a two-step screening test (non-invasive test + colonoscopy), a limitation of our study is that all individuals included are FIT-positive, thus preventing a direct comparison of the serum 6-miRNA test performance with the actual performance of the FIT in a whole CRC average-risk population. In that case, a next step validation should include serum from FIT-negative individuals. Nevertheless, to partially overcome this limitation, we also assessed the accuracy of f(Hb), easily available from FITs, and we could observe how the serum 6-miRNA signature was better for detecting AA and that combining both biomarkers could improve overall performances.

On the other hand, we know that, apart from having a limited Sn for AA, the currently used FIT provides a great number of false-positives that have to undergo unnecessary colonoscopy since all the control subjects from our study were FIT-positive when they were disease-free. In that sense, we could also think in a three-step screening test (FIT + serum 6-miRNA + colonoscopy) that could help in reducing the number of false-positives obtained with the current CRC screening methods or, at least, prioritise in the colonoscopy’s list those cases that have a high probability of having an advanced neoplastic lesion in front of those with a high probability of not having it.

In addition, and looking more specifically at the analytical method of serum miRNAs, it is utterly important to take into account a number of biological factors that may influence changes in miRNAs levels that are not a direct consequence of CRC itself [32]. As we have mentioned before, the potential clinical use of circulating miRNAs as a complementary tool for CRC screening programmes is very attractive, however, it is still hampered by several technical issues that we have to bear in mind [33]. Haemolysis, that can occur during sample extraction may influence levels of specific circulating miRNAs present in plasma or serum samples due to the rupture of erythrocytes containing miRNAs [19]. Therefore, it is very important to monitor the presence of haemolysis during the pre-analytical phase as we have performed in this study. Moreover, as there is no consensus on a suitable endogenous reference miRNA to normalize circulating miRNAs levels assessed by qRT-PCR, we have used a spiked-in exogenous combined with and endogenous miRNA control to normalise differences in miRNA recovery.

## 5. Conclusions

In conclusion, here we show the results obtained in serum of a previously described plasma-miRNA signature (miR-18a-5p, miR-19a-3p, miR-335-5p, miR-15b-5p, miR-19b-3p, miR-29a-3p) [9,10] for non-invasive CRC screening purposes. Moreover, we combined this serum-miRNA signature with another non-invasive biomarker easily available from FITs, such as f(Hb), and we show that a combination of both can improve overall diagnostic performance of advanced colorectal neoplasia with respect to each one separately. This combined signature could constitute a novel non-invasive method for early detection of CRC. However, before these novel biomarkers could be routinely used in a real clinical setting, results should be further validated in a real CRC screening population with FIT+ and FIT− individuals.

## Figures and Tables

**Table 1:** Clinical features of the study subjects. AA: advanced adenomas, CRC: colorectal cancer, TNM: Tumour Nodes Metastasis classification, SD: standard deviation.

Clinico-Pathological Characteristics	Total (*n* = 213)	Control (*n* = 80)	AA (*n* = 74)	CRC (*n* = 59)
Mean Age (SD)	6186 (553)	6202 (561)	6154 (559)	6205 (543)
Gender–Number				
Male	150	55	51	44
Female	63	25	23	15
CRC features				
TNM stage–Number				
I				30
II				13
Ш				14
Ⅳ				2
Location–Number				
Proximal				18
Distal				41
AA features				
High-grade dysplasia–Number				
Yes			27	
No			47	
Villous Component–Number				
Yes			27	
No			47	

**Table 2:** Summary data of the discriminative capacity of the serum 6-miRNA signature or faecal haemoglin concentration from ROC curve analysis, and values from combination of both. AUC: area under the curve; CI: confidence interval; SN: sensitivity; SP: specificity; PPV: positive predictive value; NPV: negative predictive value. AA: Advanced adenomas; CRC: Colorectal cancer; CRCAA: advanced colorectal neoplasia.

Groups Compared	Serum 6-miRNA Signature					Faecal Hemoglobin Concentration					Serum 6-miRNA Signature + Faecal Haemoglobin Concentration				
	AUC (95% CI)	SN (%)	SP (%)	PPV (%)	NPV (%)	AUC (95% CI)	SN (%)	SP (%)	PPV (%)	NPV (%)	AUC (95% CI)	SN (%)	SP (%)	PPV (%)	NPV (%)
CRC versus Control	0.74 (0.65–0.82)	81	56	58	79	0.85 (0.78–0.91)	81	73	69	84	0.88 (0.83–0.94)	81	78	74	84
AA versus Control	0.80 (0.72–0.87)	81	63	68	77	0.65 (0.56–0.73)	81	35	54	67	0.81 (0.75–0.88)	81	69	71	79
CRCAA versus Control	0.76 (0.69–0.82)	80	57	76	62	0.73 (0.67–0.8)	80	55	75	63	0.82 (0.76–0.88)	80	68	81	66

## Supplementary Materials

The following are available online at https://www.mdpi.com/2072-6694/11/10/1542/s1, Table S1: Individual results of the logistic regression model in serum samples for each individual miRNA from the signature (adjusted by age and gender). CI: confidence interval; OR: odds ratio.
