# Supplementary material for: Analysis of A 6-Mirna Signature in Serum from Colorectal Cancer Screening Participants as Non-Invasive Biomarkers for Advanced Adenoma and Colorectal Cancer Detection
Source: Cancers (Basel). 2019 Oct 12;11(10):1542. doi: 10.3390/cancers11101542 (PMC6827108; doi:10.3390/cancers11101542)
Supplement: Supplementary file 1 [file cancers-11-01542-s001.pdf]

Supplementary Materials: Analysis of A 6-Mirna Signature in Serum from Colorectal Cancer Screening Participants as Non-Invasive Biomarkers for Advanced Adenoma and Colorectal Cancer Detection

Table S1. Individual results of the logistic regression model in serum samples for each individual miRNA from the signature (adjusted by age and gender). CI: confidence interval; OR: odds ratio.

	**CCR vs. Controls**		**AA vs. Controls**	
**Serum miRNA**	**OR(95% CI)**	*p*-value	OR (95%CI)	*p*-value
**miR-15b-5p**	1.13 (0.85-1.50)	0.40	1.27 (0.98-1.63)	0.07
**miR-18a-5p**	1.15 (0.91-1.45)	0.24	1.01 (0.89-1.33)	0.42
**miR-29a-3p**	1.30 (0.97-1.74)	0.07	1.52 (1.16-2.00)	0.002
**miR-19a-3p**	1.31 (1.02-1.68)	0.03	1.32 (1.07-1.62)	0.01
**miR19b-3p**	0.96 (0.72-1.27)	0.77	0.94 (0.76-1.17)	0.59
**miR-335-5p**	1.30 (1.01-1.62)	0.01	1.26 (1.04-1.52)	0.01
